# Functional Status in Knee Osteoarthritis and its Relation to Demographic and Clinical Features

**DOI:** 10.31138/mjr.29.4.207

**Published:** 2018-12-18

**Authors:** Faiq I. Gorial, Shams Al-Sabah Anwer Sabah, Mena Baqer Kadhim, Norhan Badri Jamal

**Affiliations:** 1 Rheumatology Unit, Department of Medicine,; 2 Medical Students, College of Medicine, University of Baghdad, Baghdad, Iraq

**Keywords:** Knee osteoarthritis, Osteoarthritis, functional status, Western Ontario and McMaster Universities Osteoarthritis Index (WOMAC) score

## Abstract

**Objectives:**

To assess the functional status in a cohort of Iraqi patients with knee Osteoarthritis (OA) and its relation to demographic and clinical features.

**Patients and methods:**

This cross-sectional study was conducted on 150 patients with knee OA diagnosed according to the American College of Rheumatology Criteria for classification knee OA. Patients’ age, gender, body mass index (BMI), smoking history, educational level, and disease duration were recorded. The Western Ontario and McMaster Universities Osteoarthritis Index (WOMAC) score was used to measure functional status of patients with knee OA.

**Results:**

A total of 150 (97 females) patients with knee OA were recruited in the study. The mean age of patients was 52.5±10.1 year and mean BMI was 30.3±6.0 kg/m^2^. The mean of total WOMAC score was 8.05±2.10 (Range 3–12). The mean WOMAC of: pain score was 3.22 ±0.76 (1-4), stiffness score was 2.05±1.01 and for functional disability score was 2.79±0.88. There was a positive significant correlation between age of the patients and severity of knee OA assessed with total WOMAC score (p=0.026). However, there was a significant negative correlation between educational level and total WOMAC score (p=0.015).

**Conclusions:**

Functional status in knee OA was impaired and there was a statistically positive significant correlation between age of the patients and severity of knee OA with functional impairment. Also, significant negative correlation was demonstrated between educational level and functional impairment.

## INTRODUCTION

Osteoarthritis (OA) is the most common chronic degenerative joint disorder and a major public health problem associated with cartilage degeneration and joint deformity, leading to joint pain and subsequent impairments in health-related quality of life (HRQoL).^1,2^ Knee OA, one of the most common forms of OA, is considered a leading cause of disability worldwide and associated with high morbidity and mortality.^3,4^ Most OA patients suffer from great changes in their activities of daily living (ADL), and approximately 25% of them have some kind of functional limitation, such as morning stiffness, reduced joint motion, crepitus, and muscle atrophy.^5^

A number of previous studies have reported significant association between severity of knee OA and functional status^6–8^ and knee pain was considered an independent predictor of disability.^9^ To the best of our knowledge, there are no studies on functional status of knee OA among Iraqi patients. The aim of this study was to assess the functional status in patients with knee OA and its relation to demographic and clinical features.

## PATIENTS AND METHODS

### Study design

This cross-sectional study was carried out from 1^st^ March–1^st^ October 2017 in Baghdad Teaching Hospital / Rheumatology outpatient clinic.

### Patients selection

A total of 150 consecutive patients with knee OA, diagnosed by a rheumatologist according to revised ACR criteria for classification of OA of the knee,^10^ were selected and included in the study. The patients who had chronic diseases (e.g., cardiac disease, liver diseases and renal disease) and inflammatory disease that may affect knee OA were excluded.

### Data collection and evaluation

The data were collected using structured questionnaire form and included age, job, weight, height, gender, and smoking status. The Western Ontario and McMaster Universities Osteoarthritis Index (WOMAC) score was used to measure the functional status. The questionnaire form was filled by personal interview with each patient. WOMAC is a self-administered health status measure that assesses the dimensions of pain, stiffness and function (either separately or as an overall). It is available in 5-point Likert, 11-point numerical rating and 100-mm visual analogue scale (VAS) formats.^11^ The WOMAC consists of 24 items divided into 3 subscales:

▪ Pain (5 items): during walking, using stairs, in bed, sitting or lying, and standing
▪ Stiffness (2 items): after first waking and later in the day
▪ Physical Function (17 items): stair use, rising from sitting, standing, bending, walking, getting in / out of a car, shopping, putting on / taking off socks, rising from bed, lying in bed, getting in / out of bath, sitting, getting on / off toilet, heavy household duties, light household duties.

The patient’s response to each question produces a score that is then summed to derive an aggregated score for each dimension and a total score (WOMAC index) that reflects disability overall.^11^

### Ethical approval and patient consent

Informed consent was obtained from each participant included in this study according to the declaration of Helsinki. Ethical approval was taken from Department of Medicine, College of Medicine, University of Baghdad and the purpose of the study was explained to each participant prior to interview and all the patients accepted to participate in the study.

### Statistics analysis

Anderson-Darling test was used to assess the adherence of continuous data for normal distribution. Age and body mass index (BMI) followed the normal distribution, while the rest of the variables did not follow normal distribution. Mean and standard deviation was used for normally distributed data, and median and interquartile range was utilized for non-normally distributed data. T-test was used to compare means between two groups in normally distributed data, while Mann-Whitney U test for nonparametric data. Chi square test was used for discrete variables to test the association, binary logistic regression analysis was used to obtain the odds ratio that describes the relationship between dichotomous variables, and multivariate logistic regression used to see if significant variable was independent. P-values smaller than or equal to 0.05 were considered significant. The statistical calculations were performed with SPSS version 21 software package and Minitab.

## RESULTS

A total of 150 (97 females) patients with knee OA were involved in the study. The mean age was 52.5+−10.1 year and mean BMI was 30.3 +−6.0 kg/^2^. The other baseline characteristics are shown in *Table 1*.

**Table 1.** Baseline characteristics of the participants.

**Variables**	**Value**
Age (years); mean ± SD (range)	52.5 ± 10.1 (6 – 80)
Gender; no. (%)	
Female	97 (64.7%)
Male	53 (35.3%)
BMI (kg/m^2^); mean ± SD (range)	30.3 ± 6.0 (17.6–57.5)
Smoker; no. (%)	30 (20%)
Education levels; no. (%)	
Primary	99 (66%)
Secondary	31 (20.7%)
College	18 (12%)
Post graduate	2 (1.3%)
Disease duration (years); median (IQR)	1 (0.4 – 3.0)
Kellgren-Lawrence scale; no. (%)	
Grade I	16 (10.7%)
Grade II	75 (50%)
Grade III	54 (36%)
Grade IV	5 (3.3%)
WOMAC score total; mean ± SD (range)	8.05 ± 2.10 (3 – 12)
WOMAC pain score; mean ± SD (range)	3.22 ± 0.76 (1 – 4)
WOMAC stiffness score; mean ± SD (range)	2.05 ± 1.01 (0 – 4)
WOMAC function score; mean ± SD (range)	2.79 ± 0.88 (1 – 4)
NSAIDs; no. (%)	27 (18%)
Analgesic; no. (%)	123 (82%)

BMI, body mass index; WOMAC, Western Ontario and McMaster Universities Osteoarthritis Index; NSAIDs, nonsteroidal anti-inflammatory drugs; SD, standard deviation; IQR, interquartile range

**Figure 1.**
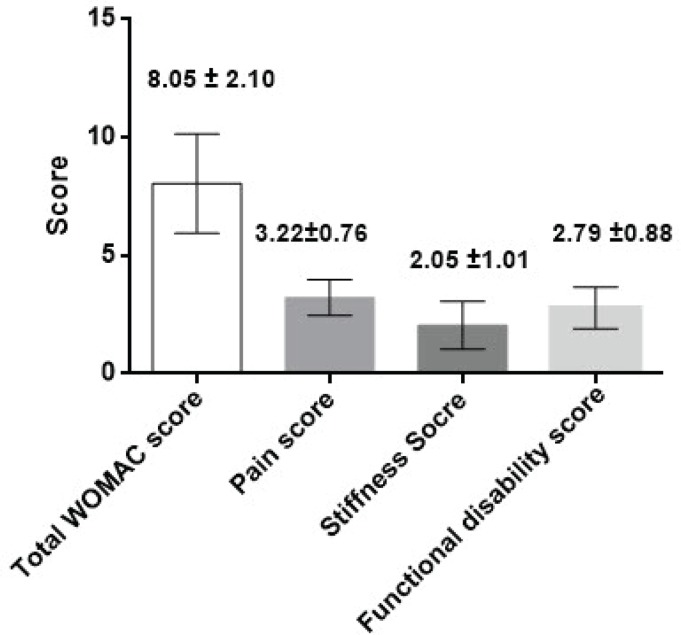
Error bar chart shows functional status in Knee osteoarthritis

The mean of total WOMAC score was 8.05 ± 2.10 (3–12). The mean WOMAC of pain score was 3.22 +− 0.76) 1–4), stiffness score was 2.05 +− 1.01 and for functional disability score was 2.79 +− 0.88 as shown in *Table 1* and *Figure 1*.

There was positive significant correlation between age of the patients, severity of knee OA measured by Kellgren-Lawrence scale with total WOMAC score. In addition, a significant negative correlation between educational level and Total WOMAC score was established (*Table 2*).

**Table 2.** Effect of baseline characteristics on Total WOMAC score using multiple linear regressions analysis.

**Variables**	**Standardized regression coefficient β**	**P value**
Gender (female)	−0.019	0.817
Age	0.185	0.026 [Sig.]
BMI	0.109	0.194
Smoking (not smoker)	0.067	0.425
Education Level (increase)	−0.203	0.015 [Sig.]
Disease Duration	0.008	0.920
Kellgren-Lawrence scale (increase)	0.319	<0.001 [Sig.]

## DISCUSSION

Knee OA is a major global burden and cause of impaired health related quality of life^12^ and associated with important economic costs and health losses.^13,14^ This is the first study assessing health related quality of life in patients with knee OA in Iraq. This cross-sectional study investigated the relationship between the functional status in patients with knee OA with the demographic and clinical features.

There was positive significant correlation between age of the patients, severity of knee OA with total WOMAC score. The quality of life in patients with knee OA is considerably impaired as a result of the chronic degenerative nature of the disease being an inevitable consequence of growing old. In OA, degradation and loss of the articular cartilage is a central feature that is sometimes attributed to “wear and tear”.^15^

The findings of the current study are in line with previous ones^1,2^ reporting that knee pain, stiffness and duration of disease may affect the functionality in patients with OA. Consequently, it would be better to consider the functional status of patients in parallel with clinical and radiological findings in daily clinical practice.^2^

Worse function was observed in elderly women suffering from OA, and the drug treatment did not result in any improvement regarding the functional status of those patients. In this context, studies on different therapeutic modalities aiming to improve function and quality of life for elderly people must be fostered.^16^

In the current study, there was a significant negative correlation between educational level and health related quality of life of knee OA (Total WOMAC score). Similar findings were reported by previous studies in which the low educational level was associated with impaired patients’ quality of life and increased symptomatic knee osteoarthris.^17,18^

This study has some limitations: most of the patients were not able to recall their symptoms retrospectively, so it is important to consider a recall bias. In addition, it was a cross-sectional study, so we could not assess the cause and effect relationship between variables and small sample. In spite of these limitations, this study was the first in Iraq to assess functional status in knee OA patients with strict inclusions and exclusions criteria.

In conclusion, functional quality of life was impaired in patients with knee OA. There was positive significant correlation between age of the patients, severity of knee OA measured by Kellgren-Lawrence scale with total WOMAC score and a significant negative correlation between educational level and Total WOMAC score. Such observations would be taken into account when treating patients with knee OA in routine rheumatology clinics.
